# Machine learning prediction of the total duration of invasive and non-invasive ventilation During ICU Stay

**DOI:** 10.1371/journal.pdig.0000289

**Published:** 2023-09-13

**Authors:** Emma Schwager, Xinggang Liu, Mohsen Nabian, Ting Feng, Robin MacDonald French, Pam Amelung, Louis Atallah, Omar Badawi

**Affiliations:** 1 Philips, Cambridge, Massachusetts, United States of America; 2 Johnson and Johnson, Rockville, Maryland, United States of America; 3 University of Maryland School of Pharmacy, Baltimore, Maryland, United States of America; Ben-Gurion University of the Negev, ISRAEL

## Abstract

Predicting the duration of ventilation in the ICU helps in assessing the risk of ventilator-induced lung injury, ensuring sufficient oxygenation, and optimizing resource allocation. Prior models provided a prediction of total duration without distinguishing between invasive and non-invasive ventilation. This work proposes two independent gradient boosting regression models for predicting the duration of invasive and non-invasive ventilation based on commonly available ICU features. These models are trained on 2.6 million patient stays across 350 US hospitals between 2010 to 2019. The mean absolute error (MAE) for the prediction of duration was 2.08 days for invasive ventilation and 0.36 days for non-invasive ventilation. The total ventilation duration predicted by our model had MAE of 2.38 days, which outperformed the gold standard (APACHE) with MAE of 3.02 days. The feature importance analysis of the trained models showed that, for invasive ventilation, high average heart rate, diagnosis of respiratory infection and admissions from locations other than the operating room were associated with longer ventilation durations. For non-invasive ventilation, higher respiratory rates and having any GCS measurement were associated with longer durations.

## Author summary

This study aimed to improve the accuracy of predicting how long a patient in the ICU will need a ventilator, which is crucial for patient safety and hospital resource management. Previous prediction models did not distinguish between invasive and non-invasive ventilation. However, our research proposes separate models for each method, which we developed using patient data from 350 US hospitals spanning nearly a decade. We used a technique known as gradient boosting regression, which leverages commonly available ICU data. Our models performed significantly better than existing standards, with errors being notably lower. Additionally, our findings highlight key factors that increase ventilation duration, including a high heart rate and a diagnosis of respiratory infection for invasive ventilation, and higher respiratory rates and any Glasgow Coma Scale (GCS) measurement for non-invasive ventilation. These models could therefore aid healthcare professionals in making better-informed decisions on patient treatment and managing their resources more effectively.

## 1. Introduction

Mechanical ventilation is a lifesaving intervention for critically ill patients in intensive care units (ICUs). Proper ventilation management aims to provide patients with sufficient oxygenation while avoiding detrimental effects such as lung injury or infection. Deciding on the optimal ventilation strategy, including ventilation mode, settings and duration of ventilation for patients can be challenging. Longer durations of ventilation can increase patient risk for ventilator-associated complications, including mortality [1] whereas delays in intubation can carry significant risk [2,3]. On the other hand, non-invasive ventilation is increasingly used to mitigate or supplement the use of invasive mechanical ventilation [4,5].

Machine learning models for benchmarking can be utilized for a variety of patient management outcomes and clinical practices such as predicting ICU length of stay, mortality and Mechanical Ventilation duration. Benchmarking the ventilation practices of an institution, which involves comparing its ventilation strategies with those of others, offers valuable insights into adherence to standards, ventilation practices, and outcomes. This is particularly relevant due to the wide range of ventilation management strategies employed by different institutions. [6,8,9]. Benchmarking is normally done through predictive models aiming to compare actual versus predicted outcomes. These predictive models can also be used for clinical decision support systems during patient care.

Several studies have utilized machine learning to predict the total duration of ventilation for patients [10–12]. One of the most widely applied models for this purpose is the APACHE (Acute physiology and chronic health evaluation] model, including its versions APACHE IVa and IVb [13,14]. These models, which use ICU patient data and are trained on data up to 2015, have provided valuable insights into the prediction of total ventilation duration.

While these models have significantly contributed to patient care, they primarily focus on the total duration of ventilation, without differentiating between invasive and non-invasive ventilation durations. Given the distinct implications and risks associated with invasive and non-invasive ventilation, as well as the increasing use of non-invasive ventilation [4,5], having specific predictive models for each ventilation type could further improve patient management strategies.

Accurate prediction of individual ventilation duration may improve patient care quality, resource planning, and patient triaging decisions. Therefore, this work aims to develop two novel algorithms to separately predict the duration of non-invasive (Model M_NIV_) and invasive (Model M_IV_) ventilation. This study utilizes one of the largest cross-country ICU databases for this purpose. The resulting models can support outcomes benchmarking as well as patient management, providing a more detailed understanding of both types of ventilation.

## 2. Materials and methods

In this study we developed two machine learning models to predict the total duration of invasive ventilation and total duration of non-invasive ventilation. The development process involved extracting patient data, defining and extracting features, model training and model performance evaluation.

### 2.1. Study population

Patient data from the Philips eICU Research Institute database (eRI database) including 3.8 million de-identified ICU patient stays from 350 US-based hospitals across more than 30 States between 2010 to 2019 was used in this study. The eRI database, captures all patient unit stays admitted to ICUs monitored by Philips eICU program with physiologic, diagnosis, and treatment information. This study was exempt from IRB oversight since there were no patient interventions due to the study’s retrospective design and since the eRI database was determined by experts at Privacy Analytics to be de-identified under HIPAA (45 Code of Federal Regulations 164.514(b)(1)) (see Table 1).

**Table 1:** The distribution of outcomes and demographics of ventilation administered. Shown are the results for ventilated patients from the full dataset (train, test, internal and external validation combined) and for stays in the training, testing, internal validation, and external validation cohorts separately. Variables with ^#^ are reported as median (IQR); variables with * are reported as mean (sd); all other variables are reported as # (%).

	**Total**	**Train**	**Test**	**Internal Validation**	**External Validation**
**Number of Patient stays**	2,655,445	1,520,838	651,381	241,455	44,883
**Ventilation duration (d)#**	2.1 (5.87)	2.11 (5.86)	2.12 (5.91)	2.11 (5.92)	1.71 (5.41)
Age (y)*	62.47 (16.15)	62.44 (16.2)	62.43 (16.21)	62.47 (16.26)	63.31 (14.38)
ICU admission BMI*	29.48 (8.52)	29.45 (8.5)	29.48 (8.57)	29.46 (8.47)	30.38 (8.66)
**Female gender**	263211 (42.4%)	159857 (42.5%)	68499 (42.4%)	25344 (42.4%)	9511 (42%)
**Hospital LOS (d)#**	8.3 (9.86)	8.25 (9.76)	8.28 (9.83)	8.27 (9.84)	9.72 (11.84)
**ICU LOS (d)#**	3.82 (5.53)	3.81 (5.5)	3.83 (5.61)	3.83 (5.56)	3.9 (5.27)

Stays with the following conditions were excluded: stays with any ventilation with an unclear mode, did not receive any ventilation, patients younger than 16 years of age, had ICU stays of less than four hours, or had missing data for the required variables (Table 2). This resulted in 2.6 M stays for each model (invasive and non-invasive duration). These stays were divided into 63%, 27%, and 10% for the training, validation, and internal test cohorts, respectively. In addition to the internal validation dataset, an external test set was used to assess the model’s generalizability to institutions with care practices not seen during model training. The external test set is part of eICU dataset and consists of a single hospital with 44K stays over the study period (2010–2019). The external test dataset was not part of the training or validation and thus represents a totally new cohort of patients that the model had not seen before.

**Table 2:** A summary of the features used as inputs to the prediction model.

Feature	Summary measure	Categorized
Admission BMI	NA	NA
Gender (1 = Female)	NA	NA
Hours in hospital prior to ICU admission	NA	NA
meanBP	Mean, variance	No
systolicBP	Mean	No
diastolicBP	Mean	No
Heart rate	Mean, variance	No
Respiration rate	Mean, variance	No
SaO2	Mean	No
Glucose	Mean	No
WBC	Mean	No
Sodium	Mean	No
Creatinine	Mean	No
Hemoglobin	Mean	No
Admission source	NA	Observation, Acute Care or Floor, Floor, Unspecified, ED, Recovery Room, Other Hospital, ER, Other ICU, Direct Admit, Chest Pain Center, PACU, ICU, SDU, OR
Admission diagnosis	NA	Yes
GCS score	NA	Yes
pH	Mean	Yes
Lactate	Mean	Yes
Albumin	Mean	Yes
PaCO2	Mean	Yes

### 2.2. Data extraction and definitions

Demographic/admission characteristics and summary measures of vital signs and laboratory measurements were extracted as input features to the models (Table 2). The model uses data from up to 24 hours after admission to predict duration among patients who are ventilated at any time during their ICU stay (including during the first 24 hours). In case, any feature is not measured for the first 24 hours, we use the data for the last 6 hours prior to ICU admission. Categorical variables such as admission source or admission diagnosis were converted to binary variables (1 if present, 0 if not). Vital signs and laboratory measurements were summarized (using mean or mean and variance) over the first 24 hours of ICU stay if available, and over the 6 hours prior to ICU admission if not. These input features are similar to some of the previous studies [15] and are selected based on the following criteria: 1) Clinically relevant as suggested by clinical experts and thus possibly predictive of the patient ventilation outcome and 2) Widely available/commonly measured and recorded patient data across hospitals. Less-commonly measured continuous variables such as lactate or pH were converted to categorical variables, including a ‘missing’ category (S1 Text).

For each patient, we used the records to identify invasive ventilation as any ventilation involving the insertion of a tube into the patient’s airway. Similarly, we identified non-invasive ventilation when no tube was inserted in the airway, but rather ventilation was performed with non-invasive techniques such as the use of mask on the nose and face (ex. BiPAP). Consistent with similar works, such as APACHE [6], the use of cPAP was not considered as any type of mechanical ventilation. A more detailed explanation on the definition and extraction of invasive and non-invasive ventilation data are provided in the supplementary section (S2 Text).

### 2.3. Model Development and training

For model development, we chose gradient-boosting regression as our machine learning framework, implemented via the XGboost package (version 1.4.2) in Python [16]. This method was chosen due to its ability to capture non-linear relationships and interactions between features in addition to model interpretability. Two models were trained using this framework: one for predicting the duration of invasive ventilation (M_IV_), and the other for predicting the duration of non-invasive ventilation (M_NIV_). Both models used all the features listed in Table 2.

Before training, we partitioned our data into training (63%), validation (27%), and internal testing sets (10%). The models were trained on the training set, with the validation set being used to tune hyperparameters and prevent overfitting. Hyperparameter tuning was done using grid search with cross-validation on the training data. This included tuning the maximum depth of decision trees (ultimately set to 10) and the number of estimators (set to 250) based on their performance on the validation set.

In order to limit the impact of outliers on model performance, we capped predictions at 10 days. If a model predicted a duration longer than 10 days, it was reported as 10 days.

### 2.4. Model evaluation

The performance of each model was evaluated using mean absolute error (MAE). We compared the performance of the new models with the predictions from APACHE IVa and APACHE IVb that are used to predict the total ventilation duration. APACHE IVa and APACHE IVb predictions for this patient cohort were automatically generated by the APACHE API and we used the outputs for comparison. To this end, the predicted total duration from the new models was calculated as the sum of the predicted duration of invasive and non-invasive ventilation models. Feature importance for the new models was evaluated using SHAP (SHapley Additive exPlanations) values [17].

## 3. Results

### 3.1 Cohort characteristics

Among the stays used to develop the duration predictions, receiving invasive ventilation was substantially more common (~600K stays received invasive ventilation; ~260K received non-invasive ventilation). Stays in the invasive ventilation cohort had longer durations (median 2.09 days vs. 1.33 days) and higher mortality (ICU mortality 13.5% vs. 8.7%). Patients in the non-invasive ventilation cohort were slightly older (Mean of 66.7 years vs. 62.5 years).

Over time, there was a slight decrease in the duration of both types of ventilation received (S1 Fig). The decrease in invasive ventilation was more noticeable, going from a median duration of 2.7 days in 2010 to 2.15 days in 2019 per ICU. There was also a wide variability in the ICU prevalence of each type of ventilation. Invasive mechanical ventilation was generally more common in all years, but the proportion of non-invasive ventilation increased over time, from 2010 to 2019, while the prevalence of invasive ventilation remained fairly constant across time. Additionally, there was significant ICU-level variation for both types of ventilation, with some ICUs typically having very short ventilation durations and others typically having extended durations.

### 3.2. Model performance

We evaluated the model performance on the validation set, internal test, and external test sets. Each model (M_IV_ and M_NIV_) was first evaluated separately to assess how well it predicted ventilation duration of each type. We also combined the predictions from the two models to compare their predictions of total ventilation duration with the predictions of APACHE IVa and APACHE IVb.

**Fig 1:**
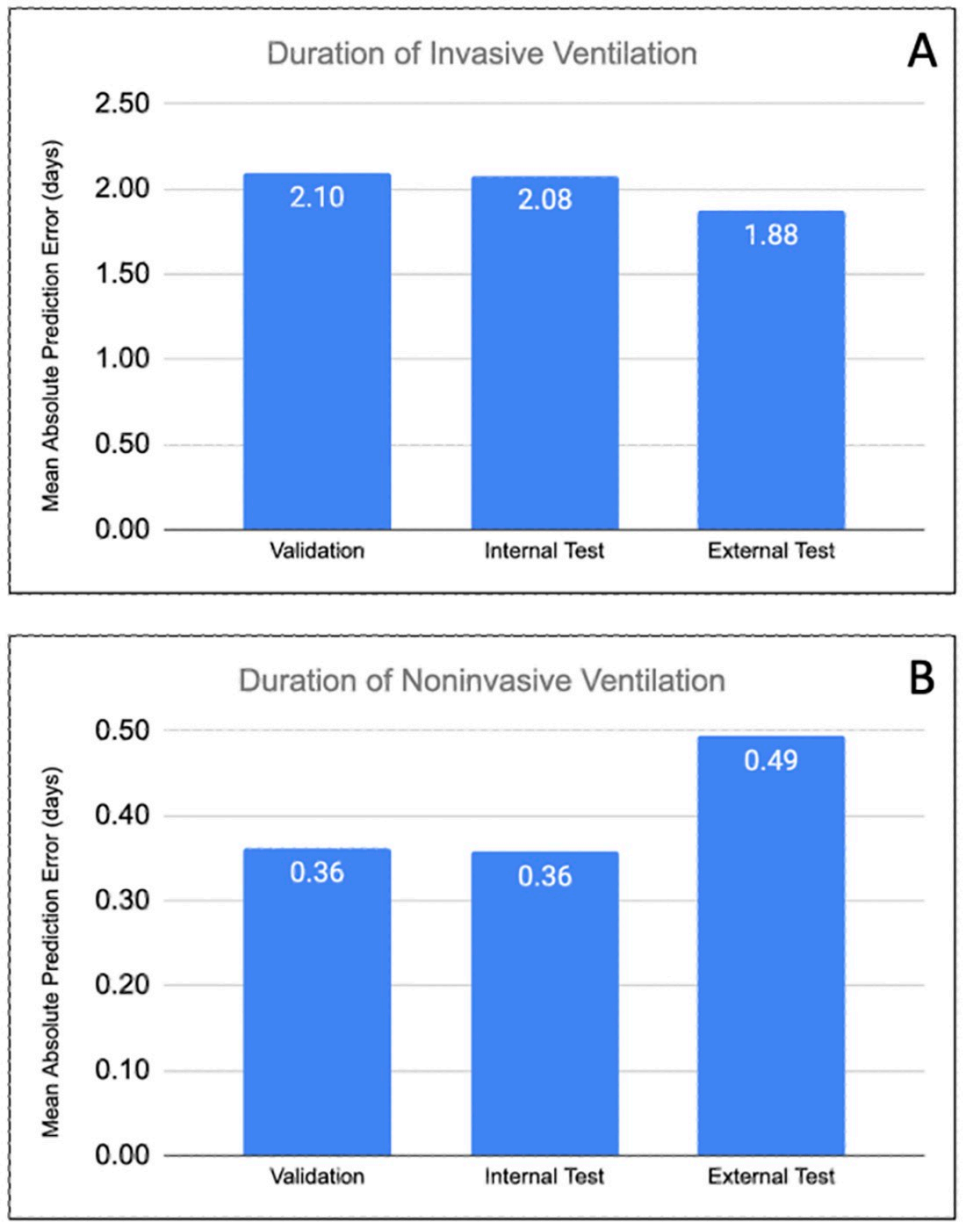
Performance of the invasive (top) and non-invasive (bottom) duration prediction models. Performance is measured by mean absolute error (MAE) and error is defined as the difference between prediction and true duration. These model accuracy evaluations are done on validation, internal validation and external validation dataset.

**Fig 2:**
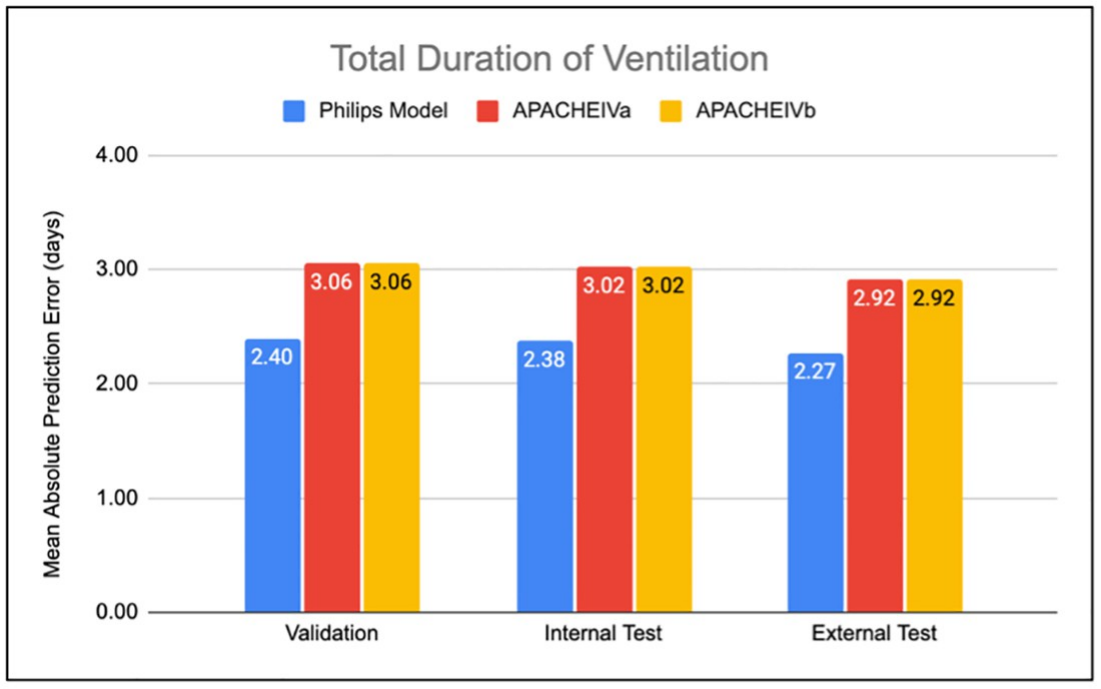
The performance of our model compared with APACHE IVa and APACHE IVb. Predictions of total ventilation duration (invasive and non-invasive) for APACHE IVa, IVb, and our models. Performance is measured by mean absolute error (MAE). Error is defined as prediction–true value. Evaluations are done on test (left), internal validation (middle) and external validation (right) dataset. Our model outperforms both APACHE models significantly.

Model prediction error, computed as mean absolute error (MAE) on the duration of invasive ventilation were 2.10, 2.08, 1.88 days on validation, internal test, and external test data respectively (Fig 1). The external test set, which is a single hospital, may not follow the distribution of the entire data and could be more on the region where model has lower error (ex. shorter duration). Similarly, the model error (MAE) on the non-invasive ventilation duration were 0.36, 0.36, 0.49 days on the validation, internal test and external test sets respectively (Fig 2).

By simply adding the duration prediction from invasive model and non-invasive model (M_IV_ + M_NIV_), we can obtain the total predicted ventilation duration for each patient. We showed that this prediction resulted in the error (MAE) of 2.40, 2.38 and 2.27 days on the validation, internal test, and external test datasets respectively. APACHE IVa and APACHE IVb models for the prediction of total ventilation duration resulted in the error (MAE) of 3.06, 3.02 and 2.92 days for the same validation, internal test, and external test sets, respectively. Comparing the results on the external test dataset, we showed **22%** improvement in the prediction of total ventilation duration over APACHE IVa and APACHE IVb models **(**Fig 2).

**Fig 3:**
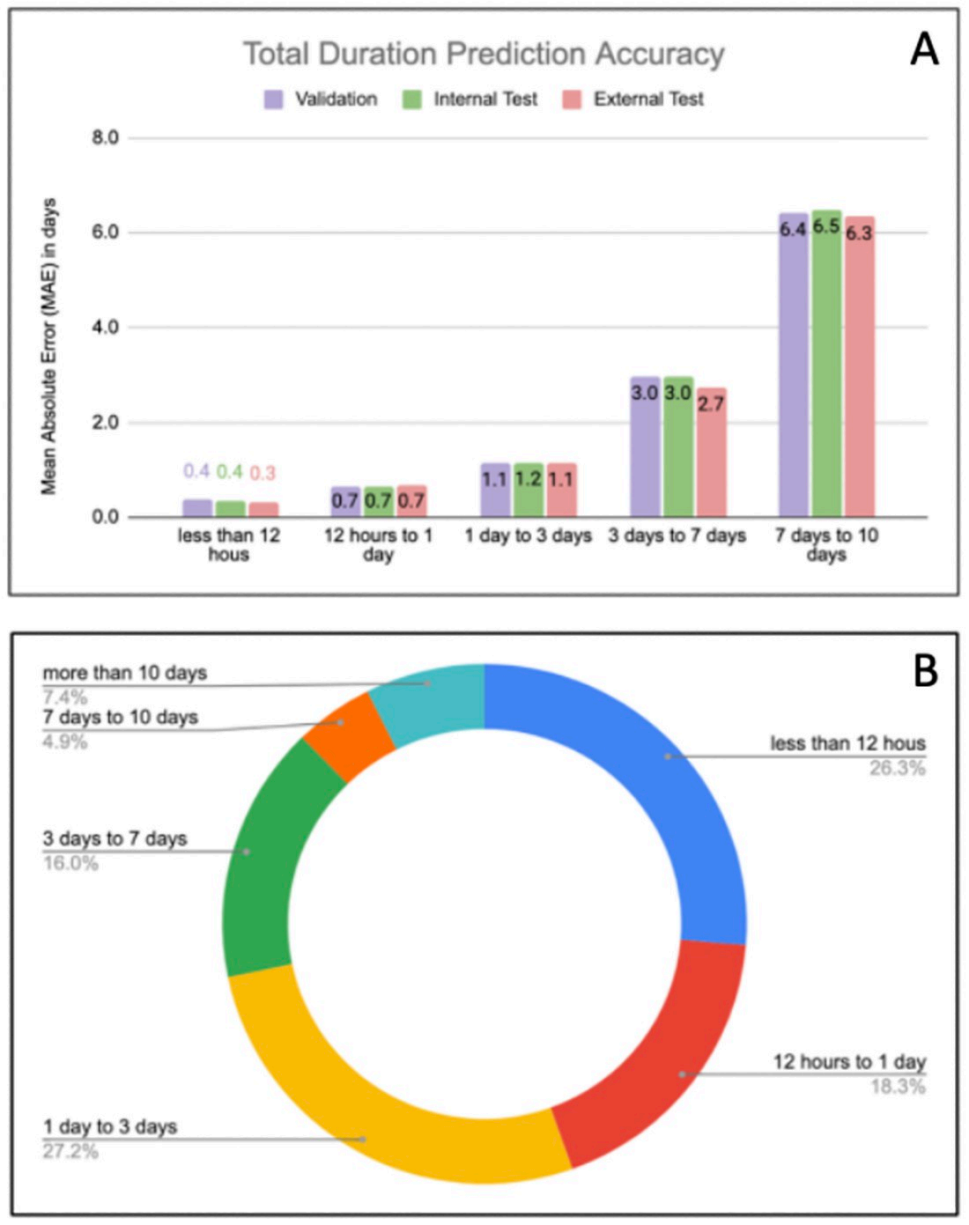
**A:** The performance of the total duration prediction model stratified by the true total ventilation duration categories. **B:** Proportion of number of patients associated with each true total duration of ventilation category.

In Fig 3, we stratified the total duration prediction error (MAE) for categories of true duration for 0.5 day, 0.5–1 day, 1–3 days, 3–7 days and 7–10 days. Similarly, we showed the same performance analysis for APACHE IVa and APACHE IVb models as illustrated in S2 Fig and S3 Fig. In addition, the proportions of patients falling in each category are also illustrated (See Fig 3A and 3B). In the category with highest number of patients (1–3 days of total ventilation), our model demonstrates the error (MAE) of **1.4 days** vs. APACHE IVa of 1.8 days and APACHE IVb of 1.8 days. The proportion of patients with 7–10 days of total ventilation are relatively small (4.9%). The models are accurate for most patients, but substantially underestimate the true duration for a smaller proportion of patients with high total duration of ventilation.

**Fig 4:**
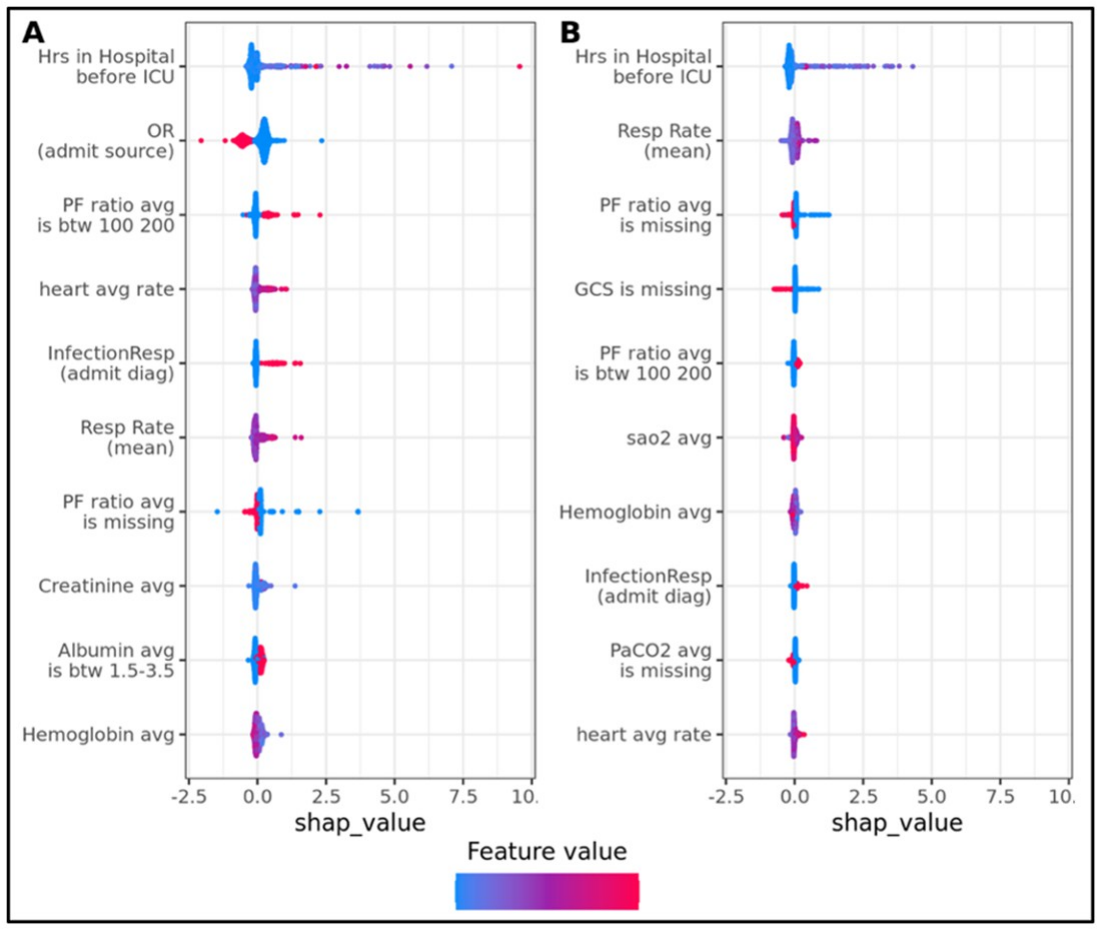
The top 10 features for predicting duration of ventilation. (**A**) Predicting duration of invasive ventilation; (**B**) predicting duration of non-invasive ventilation. Each point represents a single stay. The color represents the feature value for that stay: from the minimum (bright blue) to the maximum (bright red). The x-axis indicates the SHAP value: positive values correspond to increased duration, and negative values to decreased duration.

We computed SHAP values for the trained model to interpret the model and to examine the role of different features on the model predictions (Fig 4). SHAP values provide feature importance for each individual prediction, as well as the importance of a feature for model predictions across the entire population. This would help clinicians better understand the underlying factors and reasoning of the model predictions.

## 4. Discussion

The use of mechanical ventilation is vital to provide sufficient oxygenation for critically ill patients with respiratory failure [18]. However, the excessive use of ventilation may induce permanent lung injuries [19–21] and infection, and therefore should be avoided where unnecessary. It is also critical to utilize efficient ventilation management to optimize resources, especially when demand may significantly surpass available resources such as during pandemics. The new ventilation models predicting the duration of mechanical ventilation using patients’ information at the ICU level may contribute to addressing some of these issues. Furthermore, we are witnessing a gradual increase toward the use of non-invasive ventilation in clinical practice (S1 Fig). This highlights the importance of analyzing invasive and non-invasive ventilation separately.

Leveraging a large dataset including data from diverse hospitals with potentially varying ventilation strategies, allowed for enhanced model prediction accuracy and generalizability. The new model was trained on pre COVID-19 data (up to 2019) to better reflect current clinical practices. It is likely to provide improved accuracy compared to models trained on older datasets particularly as the number of patients with non-invasive ventilation has increased in the last few years. We intentionally excluded COVID-19 periods, as we felt that they presented unique challenges in ventilation management and it would be worth analyzing them separately.

Accuracy and performance of the models were assessed using test, internal validation, and external validation datasets. As illustrated in Fig 1, these models achieved high predictive performance, with MAE of 2.08 days for the duration of invasive ventilation and MAE of 0.36 days for the duration of non-invasive ventilation on the internal validation data. Specifying a truncation cap for the prediction outputs was implemented to improve overall accuracy and robustness to the outlier data.

While APACHE prediction models are based on linear models, the new gradient boosting models allow for capturing nonlinear interactions between patients’ features and ventilation outcomes, while maintaining model interpretability [16]. Contrary to prior models [6,22], features highly dependent on manual entry such as urinary output, active treatments, and chronic conditions were not included to improve usability across health systems.

The external test dataset may be used for robust assessment of the model as the distribution of patient data in the external test set is not necessarily similar to the aggregate distribution of the training set which comprises of patient stays from many hospitals. The model showed high prediction performances on this external test set with MAE of 1.88 days, and 0.49 days for invasive ventilation and non-invasive ventilation respectively (Fig 1).

In predicting the total duration of ventilation, our model substantially outperformed APACHE IVa and APACHE IVb (Fig 2) on the same patient population with MAE of 2.27 days for our model compared to 2.92 and 2.92 days for APACHE IVa and APACHE IVb. Our model outperformed prior research on predicting the total ventilation duration, with Sayed et al. [10] reporting RMSE of 5.87 days on part of eICU dataset and Seneff et al. [7] reporting the RMSE of 8.01 days on their validation dataset (APACHE III dataset).

We investigated the performance of the total duration model vs. APACHE models across patients, grouped by total true duration interval of ventilation (Fig 3, S2 and S3 Figs). These results indicate that our model significantly outperforms APACHE IVa and IVb models across all duration interval groups except for the short interval of < 12 hours (with MAE of 0.4 days for our model vs 0.2 days for APACHE models).

Using SHAP values [23], we investigated the features with most contribution to ventilation outcomes. For both models, the amount of time in the hospital prior to ICU admission was the strongest predictor of ventilation duration, either invasive or non-invasive (Fig 4). For invasive ventilation, admission from locations other than the operating room, high average heart rate, and an admission diagnosis of respiratory infection were associated with increased ventilation duration. For non-invasive ventilation, however, higher respiratory rate and having a measurement of GCS were both associated with increased duration.

The new models can be used both retrospectively and prospectively. Hospitals can utilize the predictions to benchmark their historical ventilation outcomes. The models can also be used prospectively as a decision support system to predict the duration of ventilation and optimize resource allocation, especially during high demand periods or peak seasons of viral diseases.

Although this model was developed on a large US-based study population, it would be important to test its performance on non-US data before deployment in other geographies, as ventilation management strategies may differ. The patient cohort did not include COVID-19 patients. Thus, it would be worth testing the model’s performance for such a patient group before deployment. The data comprised of nearly 50–50% female-male population ratio, however, it would be worth investigating the effect of racial and geographical factors among others on model bias and performance. It is also key to perform real time monitoring of prospective model performance across different cohorts over time to detect any data drift or systematic changes on the input data.

## 5. Conclusion

In conclusion, two machine learning models for predicting the duration of invasive and non-invasive mechanical ventilation were presented. To develop these models, we used a very large heterogeneous sample of US-based hospitals with automated electronic data collection of critically ill patients. We showed that our proposed ventilation models outperform APACHE IVa and APACHE IVb as well as other published models in predicting the total ventilation duration. These models can be used retrospectively as a benchmarking tool for hospitals. Further research is needed to explore if these models can also be used prospectively as clinical decision support tools for critically ill patients requiring mechanical ventilation.

## Supporting information

S1 Fig The prevalence of the Ventilation use over time from 2010 to 2019; The use of non-invasive ventilation seems to increase over time while the invasive ventilation use seems to slightly decrease. (DOCX)

S2 Fig The performance of APACHE IVa for the total duration prediction model stratified by the true total ventilation duration categories. (DOCX)

S3 Fig The performance of APACHE IVb for the total duration prediction model stratified by the true total ventilation duration categories. (DOCX)

S1 Text Variable standardization. (DOCX)

S2 Text Defining Invasive and Non-Invasive Mechanical Ventilation. (DOCX)
