# Supplementary material for: Machine learning prediction of the total duration of invasive and non-invasive ventilation During ICU Stay
Source: PLOS Digit Health. 2023 Sep 13;2(9):e0000289. doi: 10.1371/journal.pdig.0000289 (PMC10499394; doi:10.1371/journal.pdig.0000289)
Supplement: S1 Fig — (DOCX) [file pdig.0000289.s001.docx]

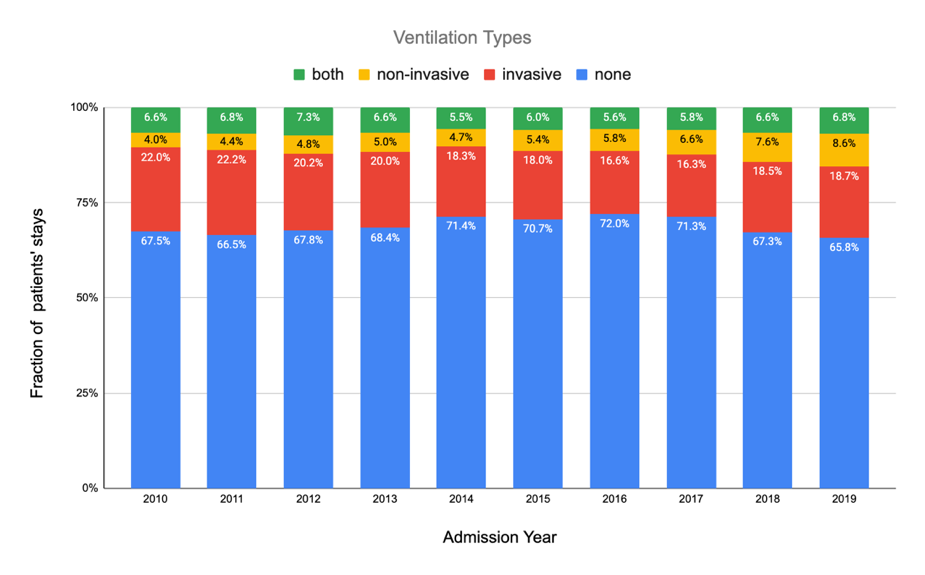


**S1 Fig*:*** *The prevalence of the Ventilation use over time from 2010 to 2019; The use of non-invasive ventilation seems to increase over time while the invasive ventilation use seems to slightly decrease.*
