# Supplementary material for: Machine learning prediction of the total duration of invasive and non-invasive ventilation During ICU Stay
Source: PLOS Digit Health. 2023 Sep 13;2(9):e0000289. doi: 10.1371/journal.pdig.0000289 (PMC10499394; doi:10.1371/journal.pdig.0000289)
Supplement: S3 Fig — (DOCX) [file pdig.0000289.s003.docx]

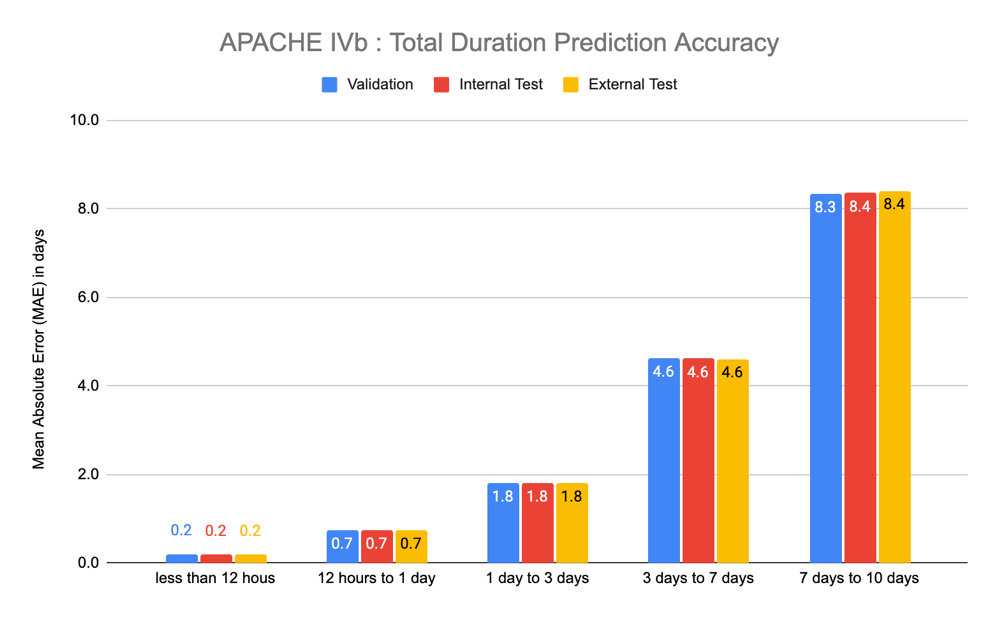


**S3 Fig*:*** The performance of APACHE IVb for the total duration prediction model stratified by the true total ventilation duration categories.
