# Supplementary material for: Machine learning prediction of the total duration of invasive and non-invasive ventilation During ICU Stay
Source: PLOS Digit Health. 2023 Sep 13;2(9):e0000289. doi: 10.1371/journal.pdig.0000289 (PMC10499394; doi:10.1371/journal.pdig.0000289)
Supplement: S1 Text — (DOCX) [file pdig.0000289.s004.docx]

## S1 Text: Variable standardization

Vital signs, laboratory data, and GCS scores were recorded in multiple ways in different sources. For example, GCS scores were recorded both in the nurse charting and the physical exam tables; within the respiratory charting table, respiratory rate could be recorded a number of different ways, including as ‘RR (patient)’, ‘Total RR’ or ‘RR Spont’.

Before combining, plausibility filters were used to remove non-physiological values. After filtering, multiple values at each timepoint within a source table were averaged. If at a single timepoint a value was available from multiple sources, a source preference order was used, based on which source was most likely to have accurate information recorded: vitalsPeriodic, vitalsAperiodic, physicalExam, nurseCharting, respiratoryCharting, lab.

Non-invasive and invasive blood pressure measurements were combined before processing.

If available, the total GCS score was used. If it was not available but the three component scores were available, it was calculated. We also included times when the GCS was unable to be scored due to the presence of medications that would alter the results. The GCS scores were binned to account for two types of missingness: unable to calculate vs. unavailable in the database.

| **Measurement** | **Normal range** |  | **Measurement** | **Normal range** |
| --- | --- | --- | --- | --- |
| Total GCS score | [1, 15] |  | Blood white blood cell | [0, 500000] |
| Eye GCS score | [1, 4] |  | Blood sodium | (0, 250] |
| Verbal GCS score | [1, 5] |  | Blood potassium | (0, 100] |
| Motor GCS score | [1, 6] |  | Blood creatinine | [0, 50] |
| Unable or estimated GCS score | [1, 5] |  | Blood hemoglobin | (0, 100] |
| Systolic BP | [0, 300] |  | Blood albumin | (0, 100] |
| Diastolic BP | [0, 250] |  | Blood lactate | [0, 100] |
| Mean BP | [0, 270] |  | Arterial blood gas, pH | [6.5, 8] |
| Heart rate | [25, 250] |  | Arterial blood gas, PaCO2 | (0, 200] |
| Respiratory rate | [0, 100] |  | Arterial blood gas, PaO2 | (0, 650] |
| SaO2 | [50, 100] |  |  |  |

**The physiological filtering ranges used.** Values within the normal range were retained. ‘(’ or ‘)’ indicates exclusion of the bound, while ‘[’ or ‘]’ indicates inclusion. For example, ‘(0, 1]’ indicates values > 0 and £ 1 were retained.

Categorizing variables: Some laboratory measurements, such as lactate or pH, are likely to be highly informative predictors, but are measured infrequently. Accordingly, we discretized such variables, using a physiologically relevant binning scheme and adding a ‘missing’ bin to indicate if the variable was missing. This allows the model to learn from the missingness of the variable without the necessity of imputation.

| **Laboratory Measurement** | **Bins for categorization** |
| --- | --- |
| Blood albumin | "<=1.5", ">1.5 - 3.5", ">3.5 - 4.0", ">4.0 - 4.5", ">4.5 - 5.0", ">5.0 - 100", "Missing" |
| Blood lactate | "<=0.5", ">0.5 - 1", ">1 - 2", ">2 - 4", ">4 - 50", "Missing" |
| Arterial blood gas, pH | "<=7.35", ">7.35 - 7.40", ">7.40 - 7.45", ">7.45 - 8.00", "Missing" |
| Arterial blood gas, PaCO2 | "<=25", ">25 - 35", ">35 - 45", ">45 - 60", ">60 - 75", ">75 - 200", "Missing" |
| Arterial blood gas, PaO2 | '<=100', '>100 - 300', '>300 - 650', 'Missing' |
| GCS score | 3, ‘>=4-6’, 7, 8, 9, 10, 11, 12, 13, 14, 15, unable_to_score, ‘Missing’ |
