# Supplementary material for: Machine learning prediction of the total duration of invasive and non-invasive ventilation During ICU Stay
Source: PLOS Digit Health. 2023 Sep 13;2(9):e0000289. doi: 10.1371/journal.pdig.0000289 (PMC10499394; doi:10.1371/journal.pdig.0000289)
Supplement: S2 Text — (DOCX) [file pdig.0000289.s005.docx]

**S2 Text: Defining Invasive and Non-Invasive Mechanical Ventilation:**

We marked mechanical ventilation as invasive when involves inserting a tube through the mouth or nose or neck into the airway to assist with breathing. Some examples include 1. Intubation which involves inserting an endotracheal tube through the mouth or nose and into the trachea, 2. Tracheotomy, involving a surgical procedure in which a small incision is made in the neck and a tube is inserted directly into the trachea.

On the other hand, we referred to a mechanical ventilation as noninvasive when a patient receives ventilation support through a mask or other device that covers the mouth and nose, rather than through a tube inserted into the airway. For example, Bilevel positive airway pressure (BiPAP) which uses a mask that fits over the nose and mouth and provides two levels of air pressure, one for inhalation and one for exhalation.

The use of Continuous positive airway pressure (CPAP) is also common which involves using a mask over the nose and mouth and delivers a constant flow of air to keep the airways open. However, consistent with similar works such as APACHE (Zimmerman, Kramer, McNair, Malila, et al., 2006), we did not consider the use of CPAP as any type of mechanical ventilation.

Based on the above definitions, for each patient, we extracted the type and the duration of invasive and non-invasive ventilation from eICU records. These records include eICU physicians direct report about the patient ventilation status as well as the data table automatically generated by the ventilator machines.
